# Identification and characterization of *Capsicum* mutants using, biochemical, physiological, and single sequence repeat (SSR) markers

**DOI:** 10.1016/j.jgeb.2024.100447

**Published:** 2024-12-02

**Authors:** Nazarul Hasan, Sana Choudhary, Neha Naaz, Nidhi Sharma, Shahabab Ahmad Farooqui, Megha Budakoti, Dinesh Chandra Joshi

**Affiliations:** a Cytogenetic and Plant Breeding Laboratory, Aligarh Muslim University, Aligarh 202002, India; b DAV University, Jalandhar, Punjab 144012, India; c Department of Plant Physiology, GBPUAT, Pantnagar 263145, India; d ICAR-Vivekananda Parvatiya Krishi Anusandhan Sansthan, Almora 263601, UK, India

**Keywords:** *Capsicum annuum* L., Capsaicin, Micronutrients, Protein, SSR markers, Mutagens

## Abstract

Identification and characterization of crop mutants through molecular marker analysis are imperious to develop desirable traits in mutation breeding programs. In the present study, macromolecular variations with altered morphological, quantitative, and biochemical traits were generated through chemically induced mutagenesis via alkylating agents and heavy metals. Statistical analysis based on quantitative traits indicating enhanced mean value in mutant lines selected from the M_4_ generation as compared to previous generations. Identification and characterization of morphology in selected mutant lines are based on altered phenotypes (e.g. tall and dwarf mutant with high yield, fruits with thick texture and bold seeds, etc.) in comparison to control populations. The useful mutations were recorded in phytochemicals (e.g. capsaicin and dihydrocapsaicin) and macro and micro nutrients profile (e.g. protein, iron, copper, cadmium and zinc) in selected mutant lines of *Capsicum annuum* L. Single Sequence Repeats (SSRs) markers analysis in selected mutant lines revealed genetic diversity in *Capsicum. annuum* L. The total of 44 alleles were observed with average number of allele 4.00. The Unweighted Pair Group Arithmetic Mean Method (UPGMA) showed maximum dissimilarity was recorded between mutant A-III and F-III followed by mutant G-III and C-III, while mutant B-III and G-III showed the lowest dissimilarity to each other followed by mutant L-III and mutant J-III. Correlation and Principal Component Analysis (PCA) revealed genetic diversity among mutant lines indicating their prioritization over other traits in indirect selection and also revealed that mutants treated with lower and medium concentrations were divergent. These mutant lines could be suitable in crop improvement programs for the broadening the genetic base of *C. annuum* L. Hierarchical Cluster Analysis (HCA) grouped the mutants into two clusters with variable euclidean distance indicated heterogeneous mutant lines developed from induced mutagenic treatments. Thus beneficial mutations could be induced in chilli genotypes via mutation breeding to enhance genetic variability in limited resources, period, and efforts.

## Highlights

• Chemically induced mutagenesis has great importance in plant breeding programs whereby random or selectively changes in genetic material may result in a desirable one.
• Ethyl-Methane Sulphonate (EMS), Methy-Methane sulphonate, Cadmium nitrate [Cd(NO_3_)_2_] and Lead nitrate [Pb(NO_3_)_2_] are the chemicals used to induce mutations at loci that regulate economically important traits.
• The study is carried out to evaluate the effects of EMS, MMS, Cd(NO_3_)_2_ and Pb(NO_3_)_2_ on changes in morphological and quantitative traits, and to understand genetic diversity through SSR markers analysis in chilli cultivar.
• In this experimental study, we are confident that chilli plant test system is suitable for testing of EMS, MMS, Cd(NO_3_)_2_ and Pb(NO_3_)_2_ to induce genetic diversity.
• Investigations are evident that the selection of mutants may be more effective by coupling the genetic heritability with genetic advance.
• Gene action is recommended for further study to select the yield contributing traits; hence, chemical mutagens have a very important role in the improvement of quantitative traits in crop breeding programs.
• Stability in genetic heritability should be analyzed and improved mutants should be hybridizing to produce the elite genotypes in the next generations of chilli cultivars.

## 1 Introduction

Chilli (*Capsicum annuum* L.) has played a significant role in food security of the world as a major vegetable and spice crop.^1^ *Capsicum* is a genus belonging to Solanaceae family. Most members of the genus are grown throughout the world as a vegetable and spice in tropical and sub-tropical regions.^2^ Genus *Capsicum* contains over 100 species and even more, botanical varieties,^3^ comprising five domesticated species (such as *C. annuum*, *C. baccatum*, *C. frutescence*, *C. pubescence*, and *C. chinense*), all believed to have originated from New world.^4^ Chilli fruit and vegetables not only have nutritional value, but also are highly effective in fighting against neuro degenerative dieseases. Chilli is an excellent source of natural antioxidants that contain a wide variety of bioactive substances which are important for preventing free radical formation through scavenging or promoting their decomposition.^5^ Chilli is one of the most widely consumed vegetables because of their combinations, color, flavor, taste, and nutritional value. Apart from being a rich source of vitamin C, chilli has vitamins A and E, a low amount of proteins, fats, carbohydrates, and traces of minerals.^6^ Capsaicin has great medicinal value and has been evaluated in the treatment of painful conditions such as rheumatic diseases, cluster pains, painful diabetic neuropathy, postherpetic neuralgia, etc ^7^ Studies conducted recently have revealed that capsaicinoids can effectively treat a number of disorders affecting sensory nerve such as arthritis, cystitis, HIV and others.^8^

Induce mutagenesis is a quick, cost-effective, robust, and proven mutation breeding approach to accelerate the process of developing and selecting novel economic traits in various crops (FAO/IAEA). The amount of genetic variation is essential for the selection of various crops,^9^ and the success of the selection of crop improvement exclusively depends on the selection process.^10^ A chemical mutagen has mild effects on plant material as reported by Oladosu et al ^11^ An advantage of chemical mutagens is that they can be applied without complicated equipment or facilities. A large number of chemical compounds are available in which a small number of chemicals have been used as mutagens tested on plants,^12^ and only restricted alkylating agents used in mutation breeding. Over 80 % of crop varieties reported to developed by using chemical mutagens, especially alkylating (IAEA, 2015), obtained via chemical mutagenesis were induced by alkylating agents. Among alkylating agents, three chemical compounds are significant alkylating mutagens: ethyl-methane sulphonate (EMS), 1-methyl-1-nitrosourea (MNU), and 1-ethyl-1-nitrosourea (ENU) and accounted to 60 % plant varieties have developed from these three alkylating agents.^12^ Heavy metals induce mutagenic effects in plants as well as animals and may result in deleterious meiotic and mitotic effects. These heavy metals altered the pattern of recombination in both model and crop plants and affected cellular processes of meiosis.^13^ Heavy metals may be used as mutagens to induce phenotypic and genotypic changes in crops, and mutations could be selected as viable and useful mutants and can be recommended for mutation breeding,^14^.^15^.

Molecular markers are important tools for the identification and characterization of mutant genotypes and also for studying the organization and assessment of genomes in different crops.^16^ Various useful molecular (DNA) markers have been developed for the characterization of genetic diversity within different mutants of a single species. These mutants are characterized through the differences of nucleotide sequences because such sequences are unaffected by season, location, agricultural practices, and growth and developmental stages of the plant.^17^ The molecular markers such as Restriction Fragment Length Polymorphism (RFLP), Amplified Fragment Length Polymorphism (AFLP), Random Amplified Polymorphic DNA (RAPD) and Single Sequence Repeat (SSR) markers have developed to characterized different mutant lines of *C. annuum* L. Single sequence repeat (SSR) markers shows multi-allelic and co-dominant type of variation among mutants and represent high reproducibility.^18^ SSR markers are used for the assessment of genetic diversity, and in study of genetic mapping, gene tagging, marker-assisted selection and identification of varieties at molecular level.^19^.

Information of genetic variations of crop plant species occurring naturally or via induced mutagenesis is essential in crop improvement for the collection, conservation, and utilization of plant genetic resources. In induced mutation breeding, speed and accuracy of mutant selection depends on precise estimations of genetic divergence in isolated and selected mutant lines using different markers viz. morphological, biochemical, physiological, and molecular markers. Assessment of genetic diversity based on phenotypes in mutation breeding indicated to environmental factors might be associated with growth and developmental process which affects the morphology of a plant.^20^ Although, morphological markers are gives conventional data to characterize the mutant lines. Therefore, molecular markers are required to validate the induced genetic diversity raised in various polygenic traits viz., quantitative, nutrient and biochemical and molecular markers has no or least effects of environmental factors. Mutant population has been generated in *C. annuum* L. for functional analysis of genes and genetic resources in breeding program. A mutant populations of chilli and tomato have been generated by using EMS mutagen,[21], [22], [23] and a total of 1,650 and 5,023 mutant lines of variety ‘Maor’ and ‘Yuwol-cho’ of *C. annuum* L. were generated to characterize the genes affecting the plant architecture and flowering, respectively,^24^.^25^ Srivastava and Mangal,^26^ reported resistant line to Tobacco Etch Virus (TEV) through screening of novel resistance alleles encoding TEV resistant gene by using Induced Local Lession IN Genome (TILLING) approach. Main objective of the study is the selection of efficient concentrations/doses of mutagens which can induce useful mutation in chilli crop.

The present study focus on (i) studying the comparative changes in morphological and biochemical parameters because of the impact of the induced mutations in high-yielding mutant lines and (ii) assessment of changes in the molecular characterization of the mutant lines of *C. annuum* L. using SSR marker technique.

## 2 Material and methods

### 2.1 Plant material and field experiments

NS 1101 is an elite variety of *C. annuum* L. in several states of India. Variety NS 1101 was produced by crossing between var. PLR1 and NS 1701 DG. Eight mutant lines from one genotype were used in this study. The genotype NS 1101 was provided by the Namdhari Seed Corporation, Nawanshahr, Punjab. The characteristics of chilli variety NS 1101 were described in Supplementary Table S1. M_1_ seeds were generated from M_0_ seeds through mutagenic treatments of alkylating agents and heavy metals in an experimental field at Aligarh Muslim University, Aligarh (27° 54′ 1.3788′' N, 78° 4′ 20.2116′' E), India. The present study was conducted during the period from October 2018 to April 2022 up to the M_4_ generation. Alkylating agents such as EMS and MMS commonly used in mutation breeding while heavy metals like Cd(NO_3_)_2_ and Pb(NO_3_)_2_ have been less documented as mutagens in the improvement of the genetic material of crops.

### 2.2 Details of the 2018–2022 field trials

**Fig. 1:**
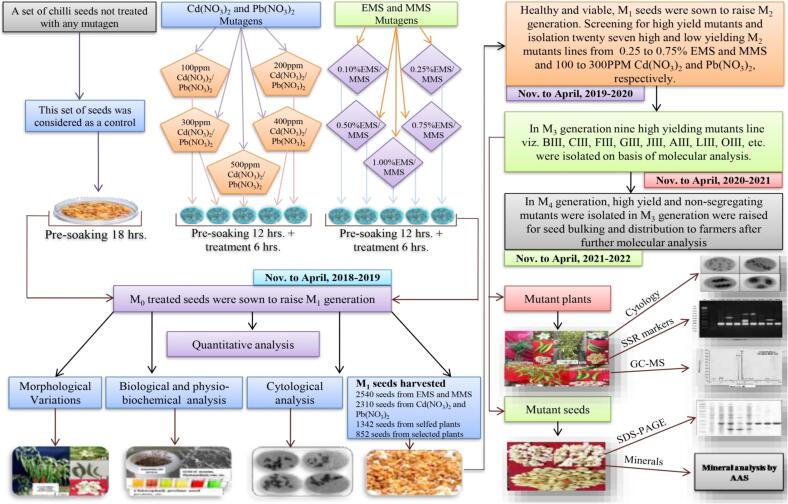
Illustration of chemical induces mutagenesis and selection processes followed during 2018–2022.

Healthy and viable seeds (10.50 %, moisture) of *C. annuum* L. were treated with different concentrations of alkylating agents [such as EMS and MMS (0.10 %, 0.25 %, 0.50 %, 0.75 % and 1.00 %)] and heavy metals [Cd(NO_3_)_2_ and Pb(NO_3_)_2_ (100 ppm, 200 ppm, 300 ppm, 400 ppm and 500 ppm)] for six hours. During mid-October 2018, the total 5,460 seeds (500 per treatment and a set of control) were sown in RCBD (Randomized Complete Block Design) design as 20 replicates of each treatment on 35.50 × 56.00 m size agriculture field at 55.00 cm inter row and 27.00 cm intra row spacing to raise M_1_ generation (Supplementary figure S1 and S2). All recommended agriculture practices such as fertilizer (nitrogen, phosphorus and potassium), irrigation and weeding were applied time to time. During first week of April 2019, all M_1_ seeds were harvested and stored to riase subsequent generations. In same year, during mid-October 2019, healthy and viable M_1_ seeds from each treatment were sown in same agriculture land to raise M_2_ populations and total 45, 340 M_2_ seeds generated from M_1_ populations in first week of April 2020. In same year, all the seeds were also sown according mutagenic treatment and out of them 34,200 seeds were survived and screen in M_3_ generation. The description of mutant lines grown/selected from M_1_ to M_3_ generation in *C. annuum* L. was represented in Supplementary Table S2. The mean data from 20 replicates on seven quantitative traits were taken. Based on quantitative analysis, 14 high and low yielding mutant lines were isolated from 0.10 %EMS, 0.25 %EMS, 0.50 %EMS, 0.10 %MMS, 0.25 %MMS, 100 ppm Pb(NO_3_)_2_, 200 ppm Pb(NO_3_)_2_ and 100 ppm Cd(NO_3_)_2_. In the first week of April 2021, all the seeds of high and low yielding mutants were harvested separately and stored for raising subsequent generations. From the M_3_ generation onwards, we advanced only high-yielding and non-segregating mutant lines to raise the M_4_ generation. In mid-October 2021, 12 healthy seeds from each high-yielding mutant plant, and the total of 2,800 seeds were sown to raise the M_4_ generation. The effective selection process led to the isolation of eight high-yielding and non-segregating mutant lines viz., CIII, BIII, AIII, GIII, LIII, NIII, JIII, and KIII in lower and medium concentrations of alkylating agents and heavy metals in M_3_ generation. The data was recorded on quantitative, biochemical, and molecular levels to develop a relative profile of each mutant line. M_4_ generation was raised for the assessment of purity of the selected mutant line by using single sequence repeat (SSR) markers and GC–MS analysis, and then seeds were distributed among the farmers. The mutagenic treatments of seeds, quantitative traits, and handling of mutant plant population-based selection were up to M_4_ generation (2018 – 2022) (Fig. 1). Eight putative mutant plants (lines) were identified and characterized based on quantitative traits with high significant yield and yield contributing traits from M_3_ populations of *C. annuum* L.

### 2.3 Morphological characterization

Morphological mutations were observed with visibly altered characters in selected mutant lines from the time of seedling emergence to plant maturity. Mutant lines were screened and isolated from induced populations and characterized into six morphological characters (plant height, leaf, branch, flower, fruit, and root) based on differential morphology. These five characters were further characterized into discrete phenotypes i.e. plant height as a tall and dwarf mutant; leaf with different shape and color; fruit with different color and texture; flower with the variable number of petals and distinct color, and root with more and fewer fibers with variable length.

### 2.4 Agro-economical study

In M_4_, data on yield and yield-contributing traits were collected from each replicate of selected mutant lines. Agro-economical traits viz. plant height, number of branches per plant, number of fruits per plant, 1000-seeds weight (g), and fresh yield per plant (g) were studied in selected mutant lines. The statistical mean value, genotypic coefficient of variation (GCV), heritability (h2), and genetic advance (GA as % of mean) was estimated (R.K. Singh and B.D. Choudhary, 1985) on the selected traits and based on these statistical analysis mutant lines indicates excellent survival and trait performance.

### 2.5 Seed protein and mineral analysis

Protein content of high yielding mutant lines was estimated by using “Bradford, Coomassie Blue G dye” method (M. M. Bradford, 1976). The content of essential minerals viz., cadmium (Cd), Zinc (Zn), Iron (Fe) and Cooper (Cu) was estimated from the seeds of high yielding mutant lines and parent genotype of *C. annuum* L. by using Atomic Absorption Spectroscopy (ASS) after wet digestion as described by (P. K. Gupta, 2004).

### 2.6 Gas Chromatography/Mass spectroscopy (GC/MS) analysis

High yielding mutant lines from M_4_ generation of *C. annuum* L. were selected to analyze phytochemicals e.g., Capsaicin and Capsaicinoids through GC–MS technique. Healthy and fresh chilli fruits were collected from high yielding mutant lines and dry under shade condition. After drying, chilli fruits were ground through mechanical blender to form a fine powder. Fine chilli powder was transferred into a sealed container having proper labeling and crude extract of samples was prepared through Soxhlet methanol extraction method. After extraction process, extract was taken into a beaker and kept on hot plate to heat at 30–40 °C until solvent become evaporated and dried extract stored in refrigerator at 4 °C. The gas chromatography-mass spectroscopy technique was applied at Biotech Park, Lucknow, to analyze the phytochemical composition in selected high yielding mutant lines. GC–MS analysis was applied followed to Pino et al., (2007) by using Thermo Scientific TSQ 8000. GC–MS was equipped with fused silica capillary column, HP-50 (30 m × 0.25 mm × 0.25 μm) and used to separate capsaicin and capsaicinoids from chilli fruits. Oven temperature was maintained at 50 °C for two minutes and then increased to 280 °C per minute. Helium (He) was used as a gas carrier and flow rate of helium was maintained to 1 mL/min. Flame ionization detector was operated as an electron impact mode of 70 eV at 230 °C for mass spectra determination (MSD). The phytochemicals composition was determine through mass spectra and retention time of know phytochemical by using automated library search on National Institute of Standard And Technology (NIST) MS search program. The identification of chemical constituents of chilli cultivars was carried out by using chemical standards in the system. The results obtained from the GC/MS analysis were represented in the form of peak area normalized (%).

## 3 Mutant characterization through molecular study

### 3.1 Lyophilization and isolation of DNA

Leaves of eight selected mutant plants were collected after 35 days of seed sowing and lyophilization was carried out to preserve their DNA in native form, overnight. Lyophilization of biological material of selected mutant plants was done by following conditions at −70°C under vacuum 10–12 miliTorr. Genomic DNA of selected mutant plants was extracted or isolated from lyophilization leaf material using slightly modified CTAB (Acetyl Trimethyl-Ammonium Bromide) (Sabouri et al. 2009). The extracted DNA of each selected mutant was confirmed by electrophoresis on 1.0 % agarose gel. Approximately 25–30 ng of DNA was used as a template in polymerase chain reaction (PCR).

### 3.2 SSR markers and PCR amplification

All experimental management practices were done as per standard protocol. The genetic diversity in selected genotypes was characterized through Simple Sequence Repeat (SSR) primers analysis at Rajeev Gandhi Center for Biotechnology (RGCB), Kerala, India.

Single Sequence Repeat (SSR) markers study was performed by following the standard protocol suggested by Temnykh et al. (2000). Selected mutant genotypes and parent genotype were subjected to polymerase chain reaction (PCR) amplification. Total eleven SSR markers were used to characterize the genetic diversity induced in high yielding QTL traits liking to their genome of selected and control genotypes. Polymerase chain reaction was performed by using Techne Thermocycler model number − TC-412 in a 10 μl reaction plate of 96 wells. The PCR kit containing of 2X PCR master mix with KAPA2G Fast DNA polymerase (used in 0.2U per 10 μl reaction), dNTPs (0.2 mM each at 1X), MgCl_2_ buffer and KAPA2 Fast PCR buffer, and forward and reverse primer were added to PCR reaction for the DNA amplification of selected and control genotypes. Polymerase chain reaction was started first denaturing of the DNA at 94 °C for 1 min followed to 35 cycle at same temperature, 55–65 °C primer annealing temperature for 1 min and 72 °C maintained for primer extension (50sec. – 1 min). After DNA amplification, PCR product was subjected to electrophoresis on 5 % polyacrylamide under aqueous solution of 0.5X TBE (Tris-Borate-EDTA) buffer at 100 V for 90 min to resolve the DNA bands. These DNA bands containing γ^32^P-AT were visualized and resolved under ultraviolet light system for the confirmation of genetic diversity with the selected and control genotypes.

### 3.3 SSR markers data analysis

DNA bands obtained by gel electrophoresis was scored as either present (1) or absent (0) in SSR markers analysis used for the genetic diversity confirmation among chilli genotypes. Molecular data of DNA bands was subjected to cluster analysis by using NTSYS statistical package (version 4.0.) to generate dendrogram based on genetic similarity/dissimilarity through Unweighted Pair Group Arithmetic Mean Method (UPGMA). The genetic diversity parameters viz., allele number, allele frequency, heterozygosity and Polymorphic Information Content (PIC) were generated by using the Power Marker software (version 3.25.) suggested by Liu and Muse (2005).

The Polymorphic Information Content (PIC) for each marker was calculated by given formula.

$${PIC}_{i}=2\times fi\times \left(1-fi\right)$$

Where, fi= (sum of alleles or bands)/no. samples.

## 4 Results

### 4.1 Morphological and quantitative characterization of mutants

**Fig. 2:**
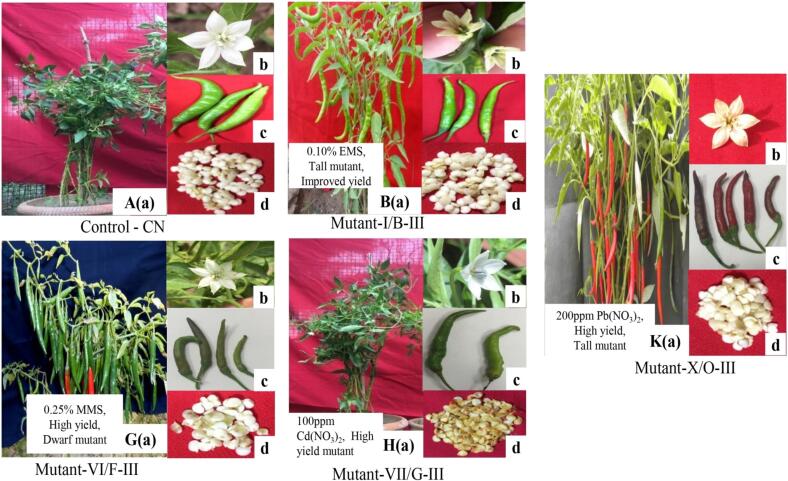
Morphological characterization of selected high yielding mutant lines of *C. annuum* L**.**

Mutagenic effects of EMS and MMS, and Cadmium nitrate and Lead nitrate [Cd(NO_3_)_2_ and Pb(NO_3_)_2_] were studied on morphological, quantitative, cytological, physiological and molecular traits in *Capsicum* mutants. Mutants with high and improved yield were represented in Fig. 2 and listed Table 1, Table 2. To assess the comparative effects of different concentrations of intra and intergenic treatment, yield and yield-attributing traits were analyzed statistically. DNA profiling and phytochemical analysis of selected mutants in M_3_ generation was also performed.

**Table 1:** Selected high/improved yielding mutants of *C. annuum* L. for molecular analysis by SSR markers (M_3_ Generation).

**S. No.**	**Control/Mutant codes**	**Mutagens used**	**Mutagenic Conc.**	**Remark**
**1.**	Control	−	−	Normal yield
**2.**	C-III	EMS	0.50 %	Tall mutant with improved high yield
**6.**	A-III	EMS	0.10 %	Tall mutant with high yield
**7.**	B-III	EMS	0.25 %	Semi-dwarf mutant with high yield
**3.**	F-III	MMS	0.25 %	Dwarf mutant with high yield
**4.**	G-III	MMS	0.50 %	Tall bushy mutant with high yield
**5.**	J-III	Cd(NO_3_)_2_	100 ppm	Tall and bushy mutant with high yield
**8.**	L-III	Cd(NO_3_)_2_	200 ppm	Semi-dwarf mutant improved yield
**9.**	O-III	Pb(NO_3_)_2_	100 ppm	Tall mutant with high yield

**Table 2:** Yield parameters in selected high yielding mutants of *C. annuum* L. (M_3_ Generation).

**S. No.**	**Mutant/Code**	**Mutagenic Conc.**	**Yield Parameters**			
			**Plant Height** **(cm)**	**No. of Branches/Plant**	**No. of Fruits/Plant**	**Fresh Yield/Plant (g)**
**1.**	Control/CN	−	78.34	11.00	30.00	93.80
**2.**	C-III	0.50 %/EMS	91.74	12.66	23.34	113.45
**3.**	A-III	0.10 %/EMS	93.00	10.00	34.65	112.34
**4.**	B-III	0.25 %/EMS	82.25	11.33	31.14	100.00
**5.**	F-III	0.25 %/MMS	53.00	11.33	29.67	90.00
**6.**	G-III	0.50 %/MMS	84.44	6.26	27.40	98.56
**7.**	J-III	100 ppm/Cd(NO_3_)_2_	94.00	12.28	31.38	102.04
**8.**	L-III	300 ppm/Cd(NO_3_)_2_	55.25	10.67	25.90	99.00
**9.**	O-III	200 ppm/Pb(NO_3_)_2_	92.63	12.60	32.44	112.33

### 4.2 Control

Plant with normal height, normal branching pattern, large dark green leaves, normal flowers with six white petals, normal white anthers, light green fruits, normal rounded white seeds, and normal yield (Fig. 2). In control, plant height, fruits per plant, and yield per plant were recorded at 83.20 cm, 29.00, and 93.60gm, respectively. The highest genetic coefficient of variance (GCV%) and genetic heritability (h2bs%) were recorded for yield per plant and fruits per plant, respectively [control] (Table 3).

**Table 3:** Statistical and genetic components analysis of yield and yield attributing traits in selected *Capsicum* mutants in M_4_ generation.

**Trait**	**Genetic** **component**	**High yielding mutant selected from M_4_ generation**								
		**CN**	**AIII**	**CIII**	**GIII**	**BIII**	**LIII**	**FIII**	**JIII**	**OIII**
**Plant height** **(cm)**	**Mean ± S.E.**	83.2^b^ **±** 0.6	93.8^a^ **±** 1.2	76.4^c^ ± 0.8	85.8^b^ ± 1.6	96.0^a^ ± 1.0	66.6^d^ ± 1.7	53.4^f^ ± 0.7	59.8^e^ ± 1.6	94.6^c^ ± 1.2
	**GCV%**	**1.67**	3.04	2.10	1.92	2.65	2.12	1.10	1.56	2.10
	**h2bs%**	45.23	47.80	51.20	48.34	56.12	53.23	61.20	45.38	50.13
	**GA%**	2.10	3.40	2.92	4.56	2.56	3.24	2.15	3.19	4.12
**Branches/plant**	**Mean**	8.6^cde^ ± 0.7	10.4^abc^ ± 1.0	6.6^e^ ± 0.9	12.8^a^ ± 0.7	6.4^e^ ± 0.5	10.0^bcd^ ± 0.9	12.4^ab^ ± 0.8	6.4^e^ ± 0.7	7.6^de^ ± 0.9
	**GCV%**	1.92	1.34	2.16	2.45	1.86	1.92	2.34	3.12	1.65
	**h2bs%**	36.67	41.23	38.80	42.12	34.56	45.78	44.32	39.10	47.45
	**GA%**	1.45	2.34	2.10	3.65	2.92	1.90	3.26	1.76	2.10
**Fruits/plant**	**Mean**	29.0^b^ ± 1.3	30.6^ab^ ± 1.3	29.6^b^ ± 0.7	31.40^ab^ ± 0.9	29.8^b^ ± 1.2	32.8^a^ ± 1.3	30.2^ab^ ± 1.0	29.8^b^ ± 1.0	31.2^ab^ ± 1.6
	**GCV%**	2.45	3.78	1.92	2.35	2.92	1.83	2.35	1.45	2.84
	**h2bs%**	51.23	56.76	61.34	58.34	52.14	59.00	62.14	45.90	51.12
	**GA%**	1.78	2.30	1.92	4.10	2.00	2.94	3.12	1.87	4.30
**1000-Seed weight (gm)**	**Mean**	6.8^ab^ ± 0.6	7.2^ab^ ± 0.6	6.6^ab^ ± 0.7	6.0^b^ ± 0.7	8.0^a^ ± 0.7	6.6^ab^ ± 0.9	6.6^ab^ ± 0.5	6.2^b^ ± 0.8	6.4^b^ ± 0.8
	**GCV%**	1.13	1.85	2.10	1.92	2.05	1.34	2.83	1.92	1.45
	**h2bs%**	26.67	32.14	28.90	36.76	31.23	38.00	41.34	28.90	34.45
	**GA%**	1.30	1.56	2.10	2.78	1.34	1.92	3.00	2.10	2.16
**Yield/plant** **(gm)**	**Mean**	93.6^d^ ± 0.7	97.6^c^ ± 1.0	102.4^b^ ± 0.7	95.8^cd^ ± 1.6	103.2^b^ ± 0.8	107.8^a^ ± 0.7	97.2^d^ ± 0.7	103.2^b^ ± 0.6	96.8^c^ ± 0.7
	**GCV%**	2.66	3.12	2.94	3.34	2.87	3.45	1.93	2.56	2.94
	**h2bs%**	49.30	54.23	59.12	55.45	61.20	65.39	55.00	57.10	50.36
	**GA%**	2.78	4.55	3.12	2.82	3.67	5.12	2.30	4.12	2.76

### 4.3 Mutant-OIII

Tall mutant with high number of fused branches, more number of small light green leaves, flowers with six creamy petals, dark green anthers, dark red fruits, light yellow large seeds, increased root length, and increased yield (Fig. 2). Plant height in mutant-OIII was recorded to be 94.60 cm, while fruits per plant and yield per plant were recorded to be 31.20 and 96.80gm, respectively, and the highest GCV% and h2bs% were estimated to be 2.94 % and 51.12 % for yield per plant and fruits per plant, respectively [200PPM Pb(NO_3_)_2_] (Table 3).

### 4.4 Mutant- BIII

Tall plants with a higher number of branches, large fruits with thick texture, light yellow flowers with six curly petals, normal seeds, and increased yield (Fig. 2). Plant height was measured to be 96.00 cm, whereas fruits per plant and yield per plant were recorded to be 29.80 and 103.20gm, respectively, and the highest values of GCV% and h2bs% were recorded at 2.87 % and 61.20 % for yield per plant [0.10 % EMS] (Table 3).

### 4.5 Mutant-FIII

Dwarf mutant with increased woody branches, dark green fruits with thin texture, light green small leaves with rounded bold seeds, late fruiting, and improved yield (Fig. 2). Mutant-FIII showed stunted growth with dwarf height and was recorded to be 53.40 cm and fruits per plant and yield per plant were recorded to be 30.20 and 97.20 g, respectively. The highest genetic coefficient of variance and genetic heritability was estimated to be 2.83 % and 61.20 % for 1000-seeds weight and plant height, respectively [0.25 % MMS] (Table 3).

### 4.6 Mutant-JIII

Dwarf mutant with woody stem and increased no of branches, larger dark green leaves, flower with white petals and free light green anthers, increased no. of dark red fruits, increased yield (Fig. 2). A plant with a height of 59.80 cm was selected as mutant-JIII and fruits per plant and yield per plant in this mutant were recorded to be 29.80 and 103.20gm, respectively. Maximum GCV% and h2bs% were recorded to be 3.12 % and 57.10 % for branches per plant and yield per plant, respectively [100PPM Cd(NO_3_)_2_] (Table 3).

### 4.7 Cytological and stomata behavior

A cytological study carried out in 45 days selected mutant plants to understand the effects of mutagens in the M_4_ generation. The chromosomal aberrations e.g. univalents, multivalents, laggards, stray chromosomes, etc., were observed at meiotic-I and meiotic-II stages in stained pollen cells under a microscope. The mutation frequency in chromosomes varies among the selected mutants and the highest mutation frequency was recorded to be 7.12 % and 6.00 % in mutant O-III and L-III at meiotic-I and meiotic-II stages, respectively. The lowest mutation frequency was 2.60 % and 1.86 % in mutant B-III at the meiotic-I and meiotic-II stages, respectively. The highest total mutation frequency in the chromosome was recorded to be 14.42 % in mutant J-III, whereas the lowest was recorded to be 4.46 % in mutant B-III (Table 4).

**Table 4:** Cytological and stomata behavior statistical analysis in selected mutant plants of *C. annuum* L.

**Mutant Code**	**Mutagen used**	**Meiotic-I**	**Meiotic-II**	**Total aberrations**	**Stomata length**	**Stomata width**	**Number of stomata**
		**Mean ± S.D.** **C.V.**	**Mean ± S.D.** **C.V.**	**Mean ± S.D.** **C.V.**	**Mean ± S.D.** **C.V.**	**Mean ± S.D.** **C.V.**	**Mean ± S.D.** **C.V.**
Control	−	0.00^f^ ± 0.00 0.00	0.00^f^ ± 0.00 0.00	0.00^f^ ± 0.00 0.00	12.56^d^ ± 0.67 5.33	2.92^bc^ ± 0.09 3.08	14.00^a^ ± 1.04 7.42
C-III	EMS	3.12^de^ ± 0.23 7.37	2.00^e^ ± 0.15 7.50	5.12^de^ ± 0.23 4.49	12.92^cd^ ± 0.72 5.57	2.40^e^ ± 0.09 3.75	13.00 ± 1.00 7.69
A-III	EMS	3.56^d^ ± 0.25 7.02	2.43^d^ ± 0.17 6.99	5.99^de^ ± 0.23 3.83	13.24^bc^ ± 0.72 5.43	2.80^c^ ± 0.10 3.57	14.00^a^ ± 1.00 7.14
B-III	EMS	2.60^e^ ± 0.21 8.07	1.86^e^ ± 0.09 4.83	4.46^e^ ± 0.21 4.70	13.00^cd^ ± 0.83 6.38	2.56^d^ ± 0.09 3.51	15.00^a^ ± 1.08 7.20
F-III	MMS	4.45^cd^ ± 0.30 6.74	2.90^c^ ± 0.12 4.13	7.35^cd^ ± 0.31 4.22	14.10^bc^ ± 0.81 5.74	3.12^ab^ ± 0.11 3.52	15.00^a^ ± 1.08 7.20
G-III	MMS	4.80^c^ ± 0.28 5.83	3.12^c^ ± 0.13 4.16	7.92^c^ ± 0.34 4.29	14.36^ab^ ± 0.92 6.40	3.00^bc^ ± 0.12 4.00	13.00^ab^ ± 0.98 7.53
J-III	Cd(NO_3_)_2_	8.90^a^ ± 0.37 4.15	5.34^ab^ ± 0.32 5.99	14.24^ab^ ± 0.32 2.24	16.00^a^ ± 0.75 4.68	3.45^a^ ± 0.12 2.47	11.00^b^ ± 0.98 8.90
L-III	Cd(NO_3_)_2_	9.10^a^ ± 0.32 3.51	6.00^a^ ± 0.21 3.50	15.10^a^ ± 0.40 2.64	16.30^a^ ± 0.78 4.78	3.12^ab^ ± 0.10 3.20	12.00^ab^ ± 0.83 6.92
O-III	Pb(NO_3_)_2_	7.12^b^ ± 0.28 3.93	5.12^b^ ± 0.18 3.51	12.24^b^ ± 0.28 2.28	15.20^ab^ ± 0.67 4.40	3.10^ab^ ± 0.10 3.22	14.00^a^ ± 0.88 6.28

**Fig. 3:**
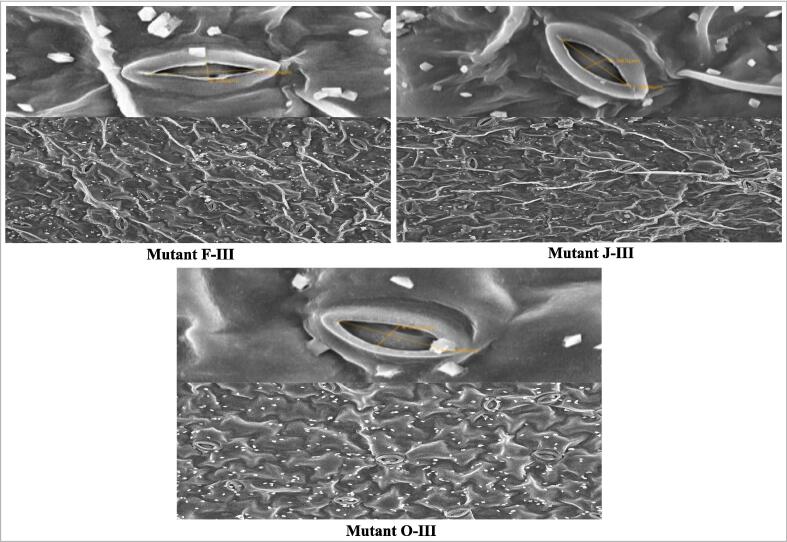
Scanning electron microscope (SEM) in selected mutants from M_4_ generation of chemical induced populations of *C. annuum* L.

Stomata study was conducted in 30-day-old mutant plants through scanning electron microscope (SEM) to understand the effects of mutagens in selected mutant plants in M_4_ generation. The parameters e.g. stomata length, stomata width, and number of stomata per leaf surface area were analyzed through a scanning electron microscope. The maximum stomata length and stomata width were measured to be 16.30 µm and 3.45 µm in mutant L-III and mutant J-III, while the lowest stomata length and stomata width were measured to be 12.92 µm and 2.40 µm in mutant C-III, respectively. The highest number of stomata per leaf surface area was measured to be 15.00 in mutant B-III and F-III, while the lowest number of stomata per leaf surface area was 11.00 in mutant J-III (Fig. 3 and Table 4).

### 4.8 Protein and mineral analysis

**Fig. 4:**
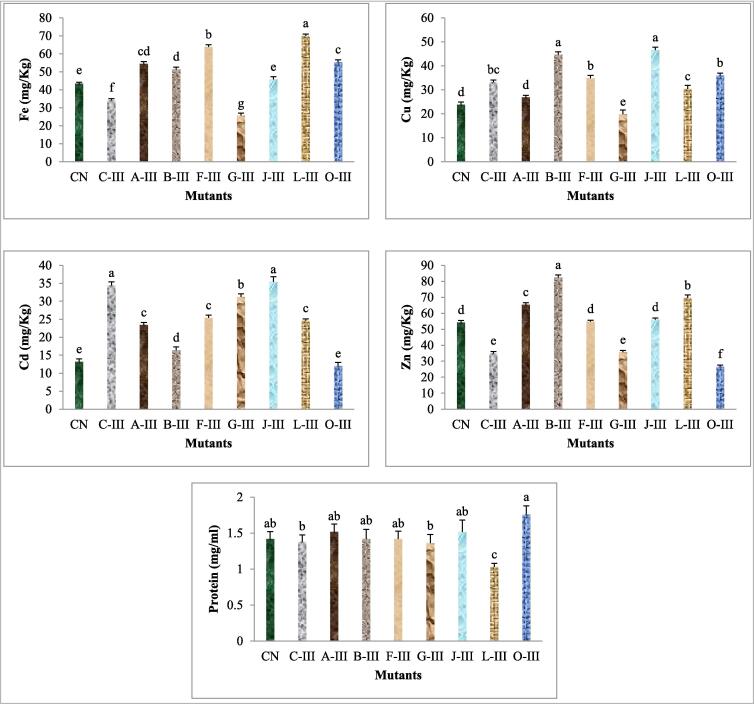
Protein and micronutrient profiling of selected mutant plants.

Estimated mean value, standard deviation (S.D.), and least significant differences (LSD) for the seed protein and mineral content (i.e. iron, copper, cadmium, and zinc) of high-yielding mutants are presented in the table. The seed protein in half of the isolated high-yielding mutants showed an insignificant mean value, while half of the mutants exhibited a significant mean value and improvement over the control plant. The highest significant increase in seed protein content (1.76 mg/ml) was recorded for the mutant O-III selected from 200 ppm Pb(NO_3_)_2_ treatment, whereas the lowest seed protein content (1.03 mg/ml) was recorded for the mutant L-III selected from 200 ppm Cd(NO_3_)_2_ treatment as compared to 1.46 mg/ml in the control. The mean value for the mineral elements in the fruits of most mutant lines showed considerable positive deviation from the control means. The maximum significant increase in the iron content (70.00 and 63.8 mg/kg) was recorded in the mutant lines L-III and F-III from 200 ppm Cd(NO_3_)_2_ and 0.25 %MMS treatments, respectively. The copper content was the highest significant (46.60 and 44.60 mg/kg) in the mutant lines J-III and B-III selected from 100 ppm Cd(NO_3_)_2_ and 0.25 %EMS treatments, respectively, as compared to 23.80 mg/kg of control mean value. The content of cadmium and zinc in mutant lines also showed variability and most of them represent significantly enhanced mean value as compared to control. The highest mean value in cadmium and zinc elements was recorded to be 35.4 mg/kg and 82.4 mg/kg in mutant J-III and B-III, respectively (Fig. 4). There were not many differences in the least significant differences (LSD) for seed protein contents between mutant lines and control plants, which implied that further improvement in seed protein cannot be expected through selection. However, differences in the mineral content of mutant lines support the possibilities of such mutants for further improvement.

### 4.9 Phytochemical analysis of *Capsicum* mutant lines

**Fig. 5:**
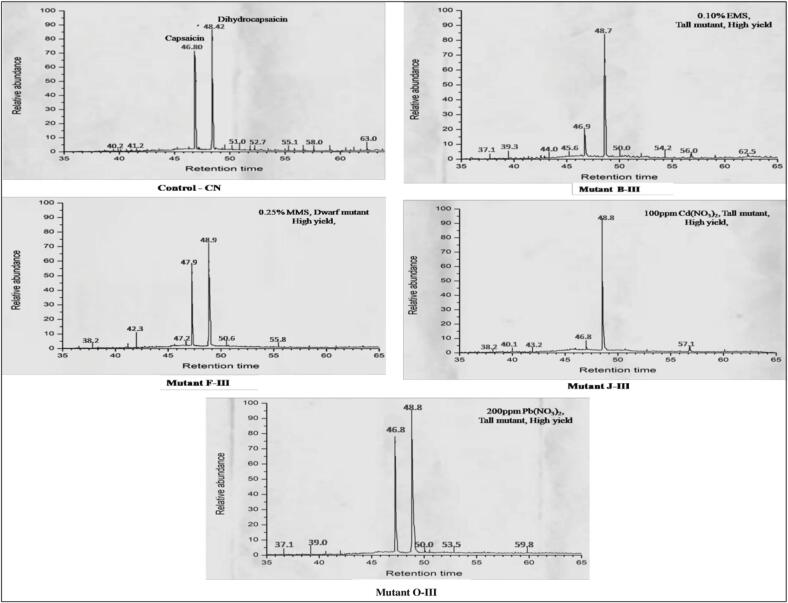
GC–MS analysis in selected high yielding mutant lines of *C. annuum* L**.**

The profile of alkaloid compounds of different chilli mutant plants was obtained by using the optimized protocol. Two CRCs (capsaicinoid-related compounds) such as capsaicin and dihydrocapsaicin in nine genotypes were indicated by the relative content and comparison through GC–MS spectra between mutants and control. In the present study, two capsaicinoids (capsaicin and dihydrocapsaicin), major contributors to chilli fruit pungency, are analyzed to determine the variations in these phytochemicals among eight high and improved yield mutants and compare them with their respective control. Phytochemical studies for capsaicin and dihydrocapsaicin in eight high-yielding lines (B-III, C-III, F-III, G-III, J-III, O-III, L-III, and AIII) and their respective control (CN) were performed through GC–MS technique. All mutants and control plants showed different peak areas of capsaicin and dihydrocapsaicin content and it is observed that the quantities of capsaicin and dihydrocapsaicin showed a great variation among the mutants as compared to the control. The highest amount of capsaicin and dihydrocapsaicin (89.00 and 93.93 %) was shown in mutant F-III and O-III while mutant L-III represents the highest amount of dihydrocapsaicin (99.34). Mutant A-III showed the lowest amount (56.23 and 73.034 %) of capsaicin and dihydrocapsaicin in comparison to their respective control (Fig. 5 and Table 5). Phytochemical analysis indicates that the mutagens have the potential to induce phytochemical changes in *Capsicum annuum* L. at different concentrations.

**Table 5:** GC–MS analysis of selected high/improved yielded mutants of *C. annuum* L. in M_3_ generation.

**S. No**	**Control/Mutant** **Code**	**Mutagens used**	**Chemical compound**	**RT** **Min:sec**	**Height** **mV**	**Area** **mV-sec**	**Amount** **% area**
**1.**	CN (Control)	−	Capsaicin Dihydrocapsaicin	46.80 48.42	178.34 204.51	345.230 945.443	78.342 90.543
**2.**	C-III	0.50 %/EMS	Capsaicin Dihydrocapsaicin	47.90 48.94	165.23 175.00	295.763 452.503	64.103 82.903
**3.**	A-III	0.10 %/EMS	Capsaicin Dihydrocapsaicin	46.83 48.87	192.45 214.34	376.921 1012.346	56.236 73.034
**4.**	B-III	0.25 %/EMS	Capsaicin Dihydrocapsaicin	46.91 48.75	182.00 193.67	765.034 478.341	83.234 54.203
**5.**	F-III	0.25 %/MMS	Capsaicin Dihydrocapsaicin	46.82 48.93	190.23 93.76	945.065 122.045	89.000 34.230
**6.**	G-III	0.50 %/MMS	Capsaicin Dihydrocapsaicin	46.75 48.80	145.03 165.40	342.872 467.341	71.763 76.003
**7.**	J-III	100 ppm/Cd(NO_3_)_2_	Capsaicin Dihydrocapsaicin	46.92 48.86	175.09 193.56	856.725 1004.762	85.675 90.364
**8.**	L-III	300 ppm/Cd(NO_3_)_2_	Capsaicin Dihydrocapsaicin	46.80 48.83	ND 218.03	ND 1432.823	ND 99.346
**9.**	O-III	200 ppm/Pb(NO_3_)_2_	Capsaicin Dihydrocapsaicin	46.78 48.93	154.45 176.34	390.347 562.651	84.094 93.932

### 4.10 Molecular analysis

High-yielding mutants of *Capsicum annuum* L. from M_3_ generation were selected and analyzed at the molecular level by using SSR markers for the detection of differences in variability among them.

**Fig. 6:**
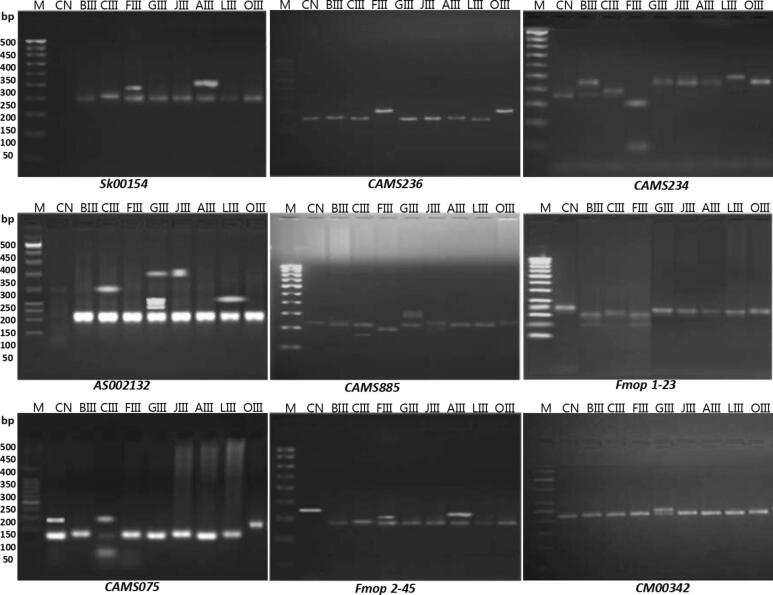
SSR amplification profile of nine primers, *Lane M:* DNA ladder with length (bp) on left. *Lane 1*–*9:* genotypes of the 9 *C. annuum* L. samples (CN III-O III).

Identification of high yield/improved mutant lines of *Capsicum annum L.* through SSR markers:

Total eleven SSR primers viz., Fmop 1–23, Fmop 1–64, Fmop 2–45, AS002132, CM00342, CAMS234, and Sk00154, etc. were used to screen nine genotypes for the amplification of chilli genomic DNA (Fig. 6). These primers showed more reproducible and highly polymorphic amplification pattern of DNA. Total 44 alleles were detected for all the seven polymorphic SSR loci with a range between 3 and 5 and average value of 4.00 alleles. The primers viz., Fmop 1–23, Fmop 2–45, CM00342, and Sk00154 has least allele number and allelic frequency ranging from 0.16 to 0.61, 0.14 to 0.52, 0.11 to 0.73, and 0.11 to 0.72, respectively. A maximum number of alleles was found to be five in other primers like Fmop 1–64, AS002132, and CAMS234 with allelic frequencies ranging from 0.09 to 0.40, 0.08 to 0.45, and 0.07 to 0.62, respectively. Genetic diversity in primers Fmop 1–23, Fmop 2–45, CM00342, and Sk00154 was measured from 0.333 to 0.322, while in primers like Fmop 2–45, CM00342, and AS0002132 was measured from 0.186 to 0.192, respectively. The total genetic diversity was measured to be 2.81 and average genetic diversity per locus was found to be 0.255 and the average polymorphic information content was measured to 0.603 (Table 6).

**Table 6:** Polymorphism information and amplification pattern of eleven primers combinations responded during SSR analysis of high yielding mutants of *C. annuum* L.

**S. R. No**	**Primer Code**	**Primer Sequences (5′-3′)**	**No of allele**	**Allele frequency**	**Genetic diversity**	**Allele size range (bp)**	**PIC**
**1.**	Fmop 1–23	F-CCAAACGAACCGATGAGTGAACA-CTC R-GACAATGTTGAAAAAGGTGGAA-GAC	3	0.23 0.61 0.16	0.33	205–260 190–234	0.563
**2.**	Fmop 1–64	F-AAGCTGAGCTGGCAAGGA-AAG R-TGAAAAGACGATTTTGTCTAAT-GCG	5	0.40 0.15 0.21 0.13 0.09	0.19	155–183 182–219	0.342
**3.**	Fmop 2–45	F-TCACCTCATAAGGGCTTTATCA-ATC R-TCCTTAACCTTACGAAACCT-TGG	3	0.34 0.52 0.14	0.33	217–255 192–230	0.535
**4.**	AS002132	F-GCTAATTACTTGCTCCGTT-TTG R-AATGGGGGAGTTTGTTT-TGG	5	0.17 0.45 0.08 0.11 0.12	0.18	164–190 200–225	0.649
**5.**	CM00342	F-CATGACCACCATGAGG-ATA R-GATAGCCACGAGCATAGT-ATT	3	0.73 0.11 0.13	0.32	210–245 215–265	0.645
**6.**	CAMS234	F-TCATGGAAAATTAACAACGC-ATA R-GGGGGTTGGAGAAAGAAAA-GTT	5	0.62 0.12 0.10 0.04 0.07	0.19	174–190 180–205	0.648
**7.**	Sk00154	F-CTACTACCGCTCCTGCT-CCT R-AGCTTCTGCTTTTGGTT-CGT	3	0.72 0.11 0.16	0.33	234–276 207–242	0.536
**8.**	CAMS075	F-ACTAATTACACATTCTGCATTTTCTC R-AGGCTCGAGTACCAGAAGA	5	0.15 0.20 0.40 0.15 0.10	0.18	190–210 170–190	0.704
**9.**	CAMS236	F-TTGTAGTTTGCTACCARRRGA R-ATGAATCCAGGGTTCCACAA	3	0.35 0.30 0.35	0.32	205–215 194–204	0.597
							
**10.**	CAMS885	F-AACGAAAAACAAACCCAATCA R-TTGAAATTGCTGAAACTCGAA	4	0.30 0.25 0.25 0.20	0.25	191–200 178–192	0.693
**11.**	CAMS855	F-AAGTGCAAGGAAGGGGACA R-CCTAACCACCCCCAAAAGTT	5	0.10 0.30 0.10 0.20 0.30	0.19	165–180 190–200	0.725
**Total**	−	−	44	−	2.81	−	6.637
**Mean**	−	−	4.000	−	0.255	−	0.603

**Fig. 7:**
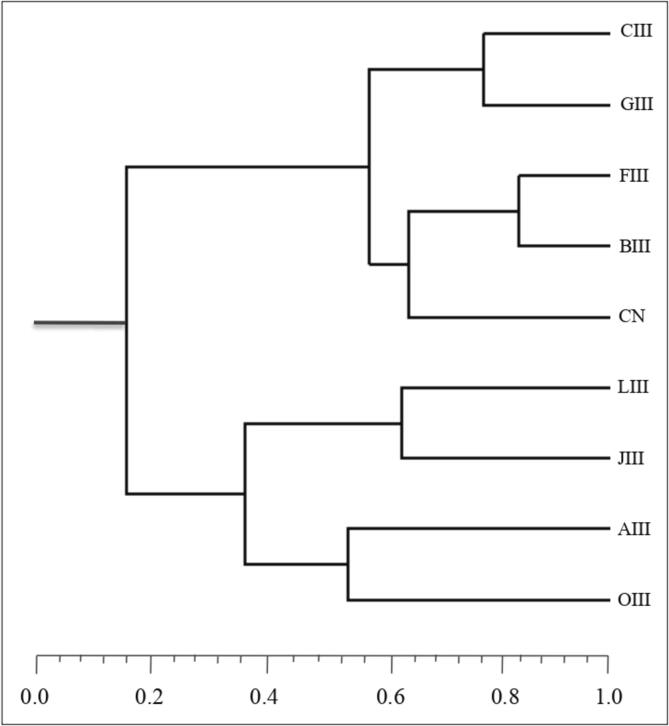
Genetic relationship among control (CN) and selected high yielding mutant lines as revealed by amplification pattern using SSR markers through Jaccard’s coefficient and UPGMA clustering method.

The comparison was made for all mutant plants including control genotypes based on SSR markers data and the distance range (Jaccard coefficient) was taken from 0.33 to 1.00. Lowest genetic dissimilarity was measured to zero which is found between the similar mutant lines (Table 7). The highest Jaccard coefficient was recorded to be 0.769 in mutant C-III and F-III followed by mutant L-III (0.750). The highest value of Jaccard coefficient in mutant J-III was found to be 0.667 and in mutant A-III was measured to be 0.615, while the lowest genetic distance in these mutants was found 0.500 and 0.364, respectively. In mutant O-III, the high Jaccard coefficient was found to be 0.615 and the lowest to 0.333. Genetic relationship among mutant plants with high yield and improved traits along with control plants was constructed in the form of a dendrogram by using binary data of SSR markers based on the unweighted pair group arithmetic mean method (UPGMA). In present study, mutant F-III (0.845) and mutant O-III (0.333) were observed to be genetically most divergent from their respective parent genotypes CN. HCA revealed that mutants and control populations were grouped into two clusters I and II. Cluster-I contains five members including control, while cluster-II comprises four members. The first cluster consisted of mutant CIII, GIII, BIII, FIII, and control, whereas the second cluster represented the mutant LIII, JIII, AIII, and OIII (Fig. 7).

**Table 7:** Distance matrix based on Jaccard Coefficient of SSR data of control and selected high/improved yielding mutants of *C. annuum* L.

	**CN**	**B-III**	**C-III**	**F-III**	**G-III**	**J-III**	**A-III**	**L-III**	**O-III**
**CN**	0.000	0.545	0.769	0.769	0.692	0.667	0.600	0.750	0.556
**B-III**		0.000	0.571	0.462	0.600	0.571	0.500	0.733	0.583
**C-III**			0.000	0.571	0.765	0.571	0.615	0.643	0.583
**F-III**				0.000	0.765	0.667	0.500	0.733	0.583
**G-III**					0.000	0.500	0.538	0.571	0.615
**J-III**						0.000	0.364	0.417	0.455
**A-III**							0.000	0.583	0.333
**L-III**								0.000	0.545
**O-III**									0.000

### 4.11 Correlation and multivariate analysis

In mutation breeding approaches, assessment of the relationship between yield and other traits is imperious. Hierarchical cluster analysis grouped mutants and control plants into different clusters. A dendrogram was constructed based on average linkage for *Capsicum* mutants and parent genotype. HCA revealed that mutants and control populations were grouped into two clusters I and II. Cluster-I contains five members including control, while cluster-II comprises four members. The first cluster consisted of the mutant CIII, GIII, BIII, FIII, and control, whereas the second cluster represented the mutant LIII, JIII, AIII, and OIII (Fig. 7).

Average value of five quantitative and six biochemical traits for two cluster groups are listed in the supplementary table 5. Results revealed that the first cluster has maximum mean value for plant height (69.5 cm), branches per plant (8.05), fruits per plant (30.15), fresh yield (101.15gm), iron (59.90 mg/kg), zinc (68.05 mg/kg) and protein (1.34 mg/ml), while second cluster represents maximum mean value for plant height (82.32 cm), branches per plant (9.80), fruits per plant (30.76), fresh yield (97.80gm), iron (40.88 mg/kg), zinc (41.48 mg/kg) and protein (1.49 mg/ml) (Supplementary Table S5).

**Fig. 8:**
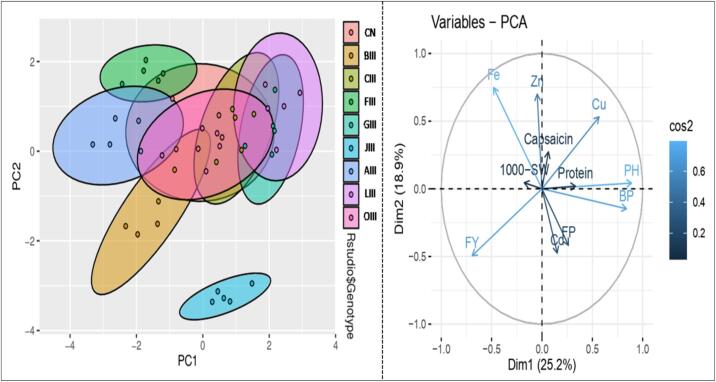
Principal component analysis and quality of representation (cos2) of yield attributing and biochemical traits in selecetd mutant lines of *C. annuum* L., high cos2 values are colored in “light blue”, mid cos2 value are colored in “blue” and low cos2 values are colored in “dark blue”. (For interpretation of the references to color in this figure legend, the reader is referred to the web version of this article.)

**Fig. 9:**
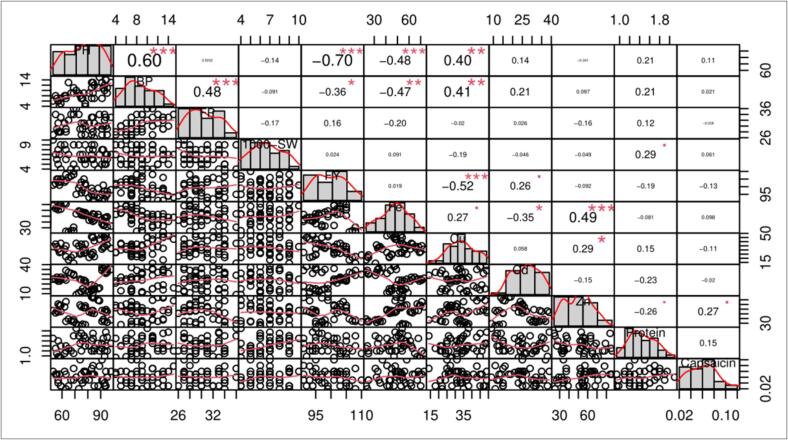
Correlation study between yield, yield attributing and biochemical traits in selected mutant lines of *C. annuum* L. PH, Plant height; BP, Branches per plant; FP, Fruits per plant; FY, Fresh yield per plant; Fe, Iron; Cu, Copper; Cd, Cadmium; Zn, Zinc; Protein; Capsaicin. Significant differences are represented as ***P < 0.001, **P < 0.01 and *P < 0.05.

Principal Component Analysis (PCA) revealed mutants viz. BIII, FIII, and JIII lie on the left side of the quadrate of the biplot, while mutants viz. OIII, LIII, GIII, CIII, and AIII lie on opposite sides in the biplot quadrate. The PCA revealed two principal components with 50.30 % variation (PC1 = 28.30 % and PC2 = 22.00 %). The magnitude of each quantitative and biochemical traits of different mutant lines represented in Fig. 8. Qualitative and biochemical traits of mutant line occur on same side represent higher value and vice versa. Cos2 value indicates the quality representation and contribution of quantitative and biochemical traits (in %) to principal components. The results revealed that FY, PH, BP, Cu, Zn, and Fe indicate good representation, while FP, 1000-seeds weight, capsaicin, protein, and Cd indicate weak representation (Fig. 8). Based on the magnitude and representation of quantitative and biochemical traits across various mutant populations, mutant LIII, JIII, AIII, and OIII formed the first cluster, whereas, in contrast, control (CN) and mutant BIII, FIII, GIII, and CIII formed second cluster. Pearson's correlation coefficient analysis helped to visualize the relationship between and among yield and yield-related traits. The results revealed a significant positive correlation coefficient relationship between yield and plant height and number of branches per plant as well as iron content. Whereas, there is no significant correlation between yield and remaining trait e.g. fruit per plant, 1000-seeds weight, Cu, Cd, Zn, protein, and capsaicin (Fig. 9).

## 5 Discussion

Mutation induction through chemical mutagens produce useful mutation with a high probability of producing dominant traits in the M_1_ generation which can be inherited in the next mutant generation,^27^.^28^ Mutation induction is the possible means to provide an enhanced level of genetic diversity among crops thus obtaining genetic variability in limited traits of a particular genotype.^29^.

Khan et al.,^30^ suggested the utilization and role of morphological markers in characterization and assessment of morphological characters of various germplasms and considered morphological traits are influenced by environmental. Different morphological changes were observed in several mutant lines at different plant growth and development stages. A mutant line with larger leaf with long petiole was observed. Manzila et al.,^31^ reported the leaf character such as leaf length and width was highly heritable and observed mutant lines with stunted growth, low branches, narrow leaves and enhanced number of corolla. Mutations in plant habit, fruits, and leaves induced by EMS were also reported in *Capsicum annuum* L.,^32^.^33^ The mutations in sepals, petals and ovule were observed^34^ and variable number of carpal and variations in flower color.^35^ The potential of the genotype-dependent mechanism of flower mutants is very useful in maintaining the genetic purity of the crop varieties in the next generation, as reported by Devi & Mullainathan,.^36^ Laskar et al.,^37^ reported that flower mutations may be correlated with the concentration or dose of the mutagens.

Enhanced quantitative traits were observed in selected mutant plants and compared with their respective control (Table 3). Karim et al.,^38^ reported quantitative changes in various chilli genotypes and suggested the association with genetic diversity. The genetic components viz., genetic coefficient of variance (GCV), heritability (h2s) and genetic advance (GA) showed variations among selected mutant plants and indicated minimum effects of environmental factors on expression of genes and significant amount of variance is considered to control by genetic composition of chilli genotypes (Table 3). Falconer and Mackay,^39^ suggested low percent of heritability at value range from 0 – 30 %, moderate at from 30 to 6-% and high heritability at more than 60 %. In the present study, broad sense heritability (h2s) was moderate for most of the traits while some traits have high value of heritability. Omolaran Bello et al.,^40^ suggested that combined heritability with genetic advance is superior to simple heritability which selected from superior individuals. High combined heritability in traits viz., number of fruits per plant and yield per plant indicates the traits are controlled by additive gene action. This standard selection procedure could be effective in selection and isolation of genotypes with desirable traits. The present study about genetic components was supported by different workers, 41 and Karim et al.,^38^ in yield per plant, and^42^ in number of fruits per plant. Yasmin et al.,^43^ reported significant increase in yield per plant and considered quantitative changes might be associated gene mutation arise in next generation. Decline in quantitative parameters could be attributed to chromosomal changes or physiological disturbances induced by mutagenic effects.^44^

A cytological study defines the specific response genotypes of mutant plants to the specific concentration or dose of a particular mutagen and also provides significant evidence for the selection of genotypes with desirable traits.^45^ In the present study, various meiotic aberrations viz., univalents, multivalents, bridges, laggard, stickiness, etc., were observed at meiosis-I and II in flower buds of selected mutant plants at different concentrations of all the mutagens (Table 4). Selected mutant plants showed the least effects of mutagens as compared to the previous generation, where, mutation frequency in chromosomes was high.

The mutations in stomata length, stomata width, and number of stomata per leaf surface area indicate changes in gaseous exchange activity and photosynthetic rate resulting enhanced productivity in selected mutant plants (Fig. 3 and Table 4). The stomata with increased size in several mutants was reported by different workers,^46^.^47^ However, stomata length and width were observed to reduce, while the number of stomata per leaf surface area was found to increase in some mutants as reported by different workers,^48^.^49^ Buckley et al.,^50^ reported the number of stomata per leaf surface area is directly related to photosynthetic rate, and stomata size and density are associated with different physiological processes.^51^.

The present study revealed that micronutrient deficiency of crops can be improved through mutation breeding. Enhanced micronutrient profiles was reported by several workers in faba bean^52^, in chickpea^53^, and in lentil.^54^ The present study revealed possibility of the combining high micronutrient with protein content and the genetic association between yield and protein content, which might be associated insignificantly, or negative correlated.^55^ The particular dose of gamma rays can induce a micronutrient profile in soybean plant.^56^ Tomlekova et al.,^57^ reported that mutation producing high beta-carotene concentrations in pepper fruits had no detrimental effects on the mineral elements and contrary to increase the bioavailability of iron and zinc in the diet. Mutation breeding for fortification of nutritional values in the major food crops has been an attractive area of crop improvement of research. Results revealed high protein content of selected mutant plants might be associated with seed yield support to results reported Kim et al., n.d.,.^12^ Chen et al.,^58^ and Laskar et al.,^54^ were reported the mutants from mutagenized populations indicated an significant increase in the protein content of seeds in comparison to their respective control plants. Non-significant improvement was reported in seed protein content in black gram,^43^ and reduces content of seed protein in *Vigna mungo*.^59^.

Methanol extraction is the most common method used to determine the phytochemicals in various crops.^60^ In the present study, only capsaicin and dihydrocapsaicin were targeted as phytochemical analysis through GC–MS techniques (Fig. 5 and Table 5). The characterization of capsaicinoids through gas chromatography-mass spectroscopy was reported by different workers Iwai et al., 2014;.^61^ Results revealed mutations in phytochemical constituents and primary sources of variations in metabolic products (Fernie et al., 2008). Younis et al.,^62^ suggested that colchicine and gamma rays increase the flavonoid concentration and antioxidant activity in the treated population as compared to the control. Arumingtyas and Ahyar,^63^ reported increased capsaicin content under the treatments of gamma radiation, and enhanced capsaicin content under 30 mM EMS and 15 mM DES treatment.^64^ These results provide insights of utilization of chemical mutagens to induce phytochemical profile of *Capsicum annum* L. Alonso-Villegas et al.,^65^ reported capsaicinoids profile of chilli extract and determined its pungency and inter and intra genetic variations induce changes in fruits pungency,^66^.^67^.

Microsatellite polymorphisms through single sequence repeat markers subjected to nine *Capsicum* genotypes were successfully amplified with the eleven SSR primer pairs. Three typical SSR marker profiles are shown in Fig. 6 (A-C) and Table 6. A total 44 alleles with an average number of 4.00 alleles per locus observed. Dhaliwal et al.,^68^ was reported 2.78 average allele value per locus, and the maximum four alleles were reported for the primer AVRDC PP32. The number of alleles detected varied from three alleles for Fmop 1–23, Fmop 2–45, CM00342, and Sk00154 to five alleles for Fmop 1–64, AS002132, and CAMS234. The size of the allele ranges from 155 bp (Fmop 1–64) to 265 bp (CM00342) (Table 4). Hossain et al.^69^ reported genetic diversity of 22 germplasm by using microsatellite markers and a total of 27 alleles with size range of 153 to 315 bp were found after gel electrophoresis. Genetic diversity within 41 genotypes of red chilli pepper recorded through eight microsatellite and total 28 alleles with average size of 3.5 allele per locus, reported by.^70^ Average number of allele per locus gives complementary information of polymorphism present in genotypes and is sufficient to co-dominant markers (IPGRI, 1999). The difference in allelic length at each locus indicates the presence of broad genetic base within the selected mutants. Haseena et al., (2008) suggested that broad genetic base induce high yield of polymorphic markers within genotypes. Average genetic diversity was recorded to be 0.255 reflecting considerable amount of polymorphism among selected mutant lines and range of genetic distance was recorded to be 0.333 to 0.769. Genetic parameters viz., genetic diversity and genetic distance were reported in several crops such as chilli,[69], [71] Tamarix^72^ and mung bean.^73^ The PIC provides an estimate to discriminate molecular markers through the number of alleles present on a locus and relative frequencies of alleles. High polymorphic information content reflected to distantly related genotypes and vice versa. Senior et al.,^74^ and Semagn et al.,^75^ were reported marker locus with average number of alleles running at equal frequencies will reflects to high polymorphic information content. Single sequence repeat (SSR) markers are very informative with high polymorphic information content (PIC > 0.50),[76], [77].^78^ The unweighted Pair Group Method with Arithmetic Mean (UPGMA) dendrogram depicted of nine *Capsicum* genotypes based on Jaccard (coefficient) genetic distance. The hierarchical cluster analysis (HCA) indicates two groups of genotypes and the highest genetic distance was recorded between the mutants A-III and F-III followed by mutant G-III and C-III (Fig. 7). In present study, mutant A-III (0.476) and mutant F-III (0.823) were observed to be genetically most divergent from their respective parent genotypes CN. This revealed that the use mutagen treatments in the present study mutated the genome of cultivar NS 1101 of *Capsicum annuum* L. Wang et al.,^79^ suggested the highest contributing clusters to the divergence should be given greater prominence to choose the cluster type for further selection and parents in subsequent hybridization. Dendrogram developed from the similarity and dissimilarity matrix of Jaccard’s coefficient values of mutants and control, showed parental genotype in separate single member cluster different from those of mutant, which confirmed that the significant amount of genetic variability was generated by mutagen treatments in the isolated high yielding mutant lines. All the nine mutant genotypes disseminated in different sub-clusters which might be possible due to higher genetic distance value (>0.6) as observed in 54 % of genotype pairs. Genetic distance value separate the genotypes in different sub-clusters where such value depend on their morphological characters of selected mutants,[80], [69].^71^

## 6 Conclusion

Results revealed mutagens are highly effective in inducing genetic variability with varying frequencies. The results decisively demonstrate the usefulness and effective potential of induced mutation breeding in the genetic improvement of *Capsicum annuum* L. for recovering the superior mutant types having enhanced yield and other variability for improved traits. Enhanced quantitative parameters indicating the mutagens were highly effective in inducing substantial inter-population genetic divergence and possibility of selection of useful traits. Molecular analysis using SSR molecular markers represents that cytomorphological, quantitative, and phytochemical changes are directly related to structural changes in chromosomes as well as DNA molecules. All mutants and control plants showed different peak areas of capsaicin and dihydrocapsaicin content indicating the mutagens have the potential to induce phytochemical changes in *Capsicum annuum* L. at variable concentrations. High-yielding *Capsicum* mutants showed a considerable level of genetic variations subjected to molecular analysis by SSR markers. Maximum dissimilarity was recorded for F-III and A-III mutants followed by O-III and B-III mutants. Principal component analysis (PCA) and correlation study revealed plant height, 1000-seed weight and number of branches per plants as primary yield components and should be on priority over other traits in direct selection process. Hierarchical clustering grouped into two clusters indicating mutagenic treatments in chilli populations induce heterogeneous mutant lines. Principal component analysis revealed mutant lines induce by lower and medium doses/concentrations were divergent and can be used in improvement program to broadening genetic base of *Capsicum annuum* L. In M_4_ generation, high yielding mutant lines were reflecting enhanced nutrient density and improved genetic gain. Considering enhanced nutrient density and improved genetic gain, further mutant characterization and multi-locations trials could be fruitful to release high-yielding and fortified chilli varieties. Mutations generated in economically important traits can be used for inheritance and genetic study of *C. annuum* L. as well.

## Funding

There is no specific funding received by any funding agency in this research.

## CRediT authorship contribution statement

**Nazarul Hasan:** Writing – original draft, Conceptualization. **Sana Choudhary:** Supervision. **Neha Naaz:** Methodology, Data curation. **Nidhi Sharma:** Visualization, Formal analysis. **Shahabab Ahmad Farooqui:** Resources. **Megha Budakoti:** Formal analysis. **Dinesh Chandra Joshi:** Writing – review & editing, Investigation.

## Declaration of competing interest

The authors declare that they have no known competing financial interests or personal relationships that could have appeared to influence the work reported in this paper.
