# Supplementary material for: Identification and characterization of Capsicum mutants using, biochemical, physiological, and single sequence repeat (SSR) markers
Source: J Genet Eng Biotechnol. 2024 Dec 2;22(4):100447. doi: 10.1016/j.jgeb.2024.100447 (PMC11652771; doi:10.1016/j.jgeb.2024.100447)
Supplement: Supplementary Data 1 [file mmc1.docx]

**Journal of Genetic Engineering and Biotechnology**

**Identification and Characterization of *Capsicum* Mutants Using, Biochemical, Physiological, and Single Sequence Repeat (SSR) Markers**

Nazarul Hasan^1*^ Sana Choudhary^1^ Neha Naaz^1^ Nidhi Sharma^1^ Shahabab Ahmad Farooqui^2^ Megha Budakoti^3^ Dinesh Chandra Joshi^4^

^1^Cytogenetic and Plant Breeding Laboratory, Aligarh Muslim University, Aligarh, India-202002

DAV University Jalandhar Punjab, India-144012^2^

^3^Department of Plant Physiology, GBPUAT, Pantnagar-263145

^4^ICAR-Vivekananda Parvatiya Krishi Anusandhan Sansthan, UK, India-263601

**Correspondence**

Dr. Nazarul Hasan

[nazarulpasha143@gmail.com](mailto:nazarulpasha143@gmail.com)

**Table S1:** Description of selected *Capsicum annuum* L

| **Variety** | **Source** | **Characters** |
| --- | --- | --- |
| NS 1101 | Namdhari seeds | Plant achieves a height of 82.0 m. The light green fruit turns into shiny deep red at maturity. Flowering time 53 – 58 days. Fruit length is 7-8 cm. Maturity is about 105 – 115 days. The fruit is highly pungent and tolerant of viruses. The yield is about 6.2 t/ha of dry pods and 26.5 t/ha green pods. |

| **Mutagen**  **MMS/EMS** | **M_1_** | | | **M_2_** | | | | **M_3_** | | | | |
| --- | --- | --- | --- | --- | --- | --- | --- | --- | --- | --- | --- | --- |
|  | **Seed Treated** | **Seeds Germinated** | **Fertile Plants** | **M_2_ Lines** | **Total M_2_ Seeds** | **Seeds Germinated** | **Fertile Plants** | **M_3_ Lines** | **Total M_3_**  **Seeds** | **Seeds Germinated** | **Fertile Plants** | |
| **Control*** | 0 | 149 | 149 | 149 | 620 | 612 | 611 | 611 | 610 | 604 | 604 | |
|  | **Ethyl-Methane Sulphonate (EMS)** | | | | | | | | | | | |
| **0.10%** | 150 | 147 | 145 | 145 | 508 | 502 | 499 | 499 | 512 | 503 | 501 | |
| **0.25%** | 150 | 145 | 142 | 142 | 500 | 492 | 488 | 488 | 505 | 500 | 495 | |
| **0.50%** | 150 | 142 | 138 | 138 | 496 | 481 | 478 | 478 | 495 | 489 | 482 | |
| **0.75%** | 150 | 138 | 133 | 133 | 485 | 470 | 468 | 468 | 488 | 482 | 476 | |
| **1.00%** | 150 | 132 | 127 | 127 | 472 | 458 | 456 | 456 | 480 | 473 | 465 | |
|  | **Methyl-Methane Sulphonate (MMS)** | | | | | | | | |  | | |
| **0.10%** | 150 | 145 | 142 | 142 | 495 | 497 | 495 | 495 | 508 | 500 | 496 | |
| **0.25%** | 150 | 137 | 133 | 133 | 486 | 482 | 480 | 480 | 500 | 494 | 492 | |
| **0.50%** | 150 | 131 | 127 | 127 | 480 | 470 | 465 | 465 | 488 | 482 | 476 | |
| **0.75%** | 150 | 124 | 119 | 119 | 465 | 458 | 454 | 454 | 480 | 472 | 469 | |
| **1.00%** | 150 | 115 | 109 | 109 | 452 | 441 | 433 | 433 | 471 | 463 | | 458 |
|  | **Lead Nitrate (Pb(NO_3_)_2_)** | | | | | | | | | | | |
| **10ppm** | 150 | 142 | 138 | 138 | 495 | 492 | 486 | 486 | 504 | 490 | 490 | |
| **20ppm** | 150 | 136 | 131 | 131 | 488 | 481 | 480 | 480 | 497 | 484 | 483 | |
| **30ppm** | 150 | 127 | 121 | 121 | 477 | 465 | 460 | 460 | 486 | 471 | 466 | |
| **40ppm** | 150 | 116 | 110 | 110 | 465 | 452 | 544 | 544 | 475 | 460 | 453 | |
| **50ppm** | 150 | 104 | 98 | 98 | 447 | 438 | 430 | 430 | 462 | 448 | 438 | |
|  | **Cadmium Nitrate (Cd(NO_3_)_2_)** | | | | | | | | | | | |
| **10ppm** | 150 | 132 | 127 | 127 | 493 | 488 | 480 | 480 | 500 | 482 | 472 | |
| **20ppm** | 150 | 126 | 118 | 118 | 488 | 476 | 468 | 468 | 491 | 472 | 463 | |
| **30ppm** | 150 | 111 | 102 | 102 | 472 | 463 | 450 | 450 | 476 | 459 | 446 | |
| **40ppm** | 150 | 95 | 86 | 86 | 456 | 446 | 435 | 435 | 462 | 442 | 433 | |
| **50ppm** | 150 | 82 | 71 | 71 | 440 | 428 | 415 | 415 | 448 | 431 | 418 | |
| **Pooled total** | **3,150** | **2,676** | **2,424** | **2,424** | **10,180** | **9,992** | **9,264** | **9,264** | **10,338** | **10,101** | **9,976** | |

**Table S2:** Description of mutant lines grown/selected from M_1_ to M_3_ generation in *C. annuum* L.

*for control set, 150 seeds were sown in same field.

| **Sl. No.** | **Traits** | **Method of measurement** |
| --- | --- | --- |
| 1 | Plant height (cm) | height from the base up to the apex of the plant |
| 2 | Days to flowering | days are taken from sowing to the date of opening of the first flower |
| 3 | Days to maturity | days are taken from sowing until the date of harvesting |
| 4 | Fruits per plant (number) | total number of fruit per plant |
| 5 | Branches per plant (number) | number of branches per plant at maturity |
| 6 | 1000-seeds weight (g) | Weight of 1000-seeds at maturity |
| 7 | Fresh yield per plant (g) | Weight of random fresh fruits |
| 8 | Dry yield per plant (g) | Weight of random dry fruits at maturity |
| 9 | Root length (cm) | Length of root from root base to root cap |

**Table S3:** List of morphological traits and description of their quantitative traits in *C. annuum* L.

| **Traits** | **Source of variation (mutants)** | **df** | **Var. NS 1101** | | |
| --- | --- | --- | --- | --- | --- |
|  |  |  | **Mean square** | **F** | **P** |
| Plant height (cm) | Between | 8 | 948.072 | 177.764 | 0.000^**^ |
|  | Within | 36 | 5.333 | 170.153 | 0.000^**^ |
| Number of branches/plant | Between | 8 | 31.472 | 9.505 | 0.000^**^ |
|  | Within | 8 | 3.311 | 10.677 | 0.000^**^ |
| Number of fruits/plant | Between | 36 | 6.706 | 0.930 | 0.504^ns^ |
|  | Within | 8 | 7.211 | 0.929 | 0.496^ns^ |
| 1000-seeds weight (gm) | Between | 36 | 2.506 | 0.968 | 0.476^ns^ |
|  | Within | 8 | 2.589 | 0.991 | 0.453^ns^ |
| Fresh yield (gm) | Between | 36 | 127.106 | 31.255 | 0.000^**^ |
|  | Within | 8 | 4.067 | 34.145 | 0.000^**^ |
| Iron (Fe) | Between | 36 | 964.450 | 140.910 | 0.000^**^ |
|  | Within | 8 | 6.844 | 139.066 | 0.000^**^ |
| Copper (Cu) | Between | 36 | 397.356 | 55.966 | 0.000^**^ |
|  | Within | 8 | 7.100 | 58.852 | 0.000^**^ |
| Cadmium (Cd) | Between | 36 | 381.900 | 89.976 | 0.000^**^ |
|  | Within | 8 | 4.244 | 102.771 | 0.000^**^ |
| Zinc (Zn) | Between | 36 | 1643.956 | 211.668 | 0.000^**^ |
|  | Within | 8 | 7.767 | 233.916 | 0.000^**^ |
| Protein (mg/ml) | Between | 36 | 0.182 | 2.757 | 0.017^*^ |
|  | Within | 8 | 0.066 | 3.141 | 0.011^**^ |
| Capsaicin | Between | 36 | 0.001 | 1.494 | 0.194^ns^ |
|  | Within | 8 | 0.001 | 0.722 | 0.654^ns^ |

**Table S4:** One-Way ANOVA results for the ten phenotypic quantitative traits of mutants in M_4_ generation. Significant difference are indicated as ‘***’ for P < 0.001, ‘**’ for P < 0.01, ‘*’ for P < 0.05, ‘**.**’ for P < 0.1 and ‘ns’ for non significance, df represent degree of freedom.

**Table S5:** Characteristic means of two cluster groups of nine genotypes of chilli

| **Characters** | **Cluster** | |
| --- | --- | --- |
|  | **Cluster-I** | **Cluster-II** |
| Plant height (cm) | 69.5 | 82.32 |
| Branch per plant | 8.05 | 9.80 |
| Fruits per plant | 30.15 | 30.76 |
| 1000-seeds weight (gm) | 6.7 | 6.72 |
| Fresh yield (gm) | 101.15 | 97.80 |
| Iron (Fe) (mg/kg) | 59.9 | 40.88 |
| Copper (Fe)(mg/kg) | 34.15 | 31.88 |
| Cadmium (Cd)(mg/kg) | 22.45 | 25.24 |
| Zinc (Zn) (mg/kg) | 68.05 | 41.48 |
| Protein (mg/ml) | 1.3475 | 1.49 |
| Capsaicin | 0.0677 | .06 |


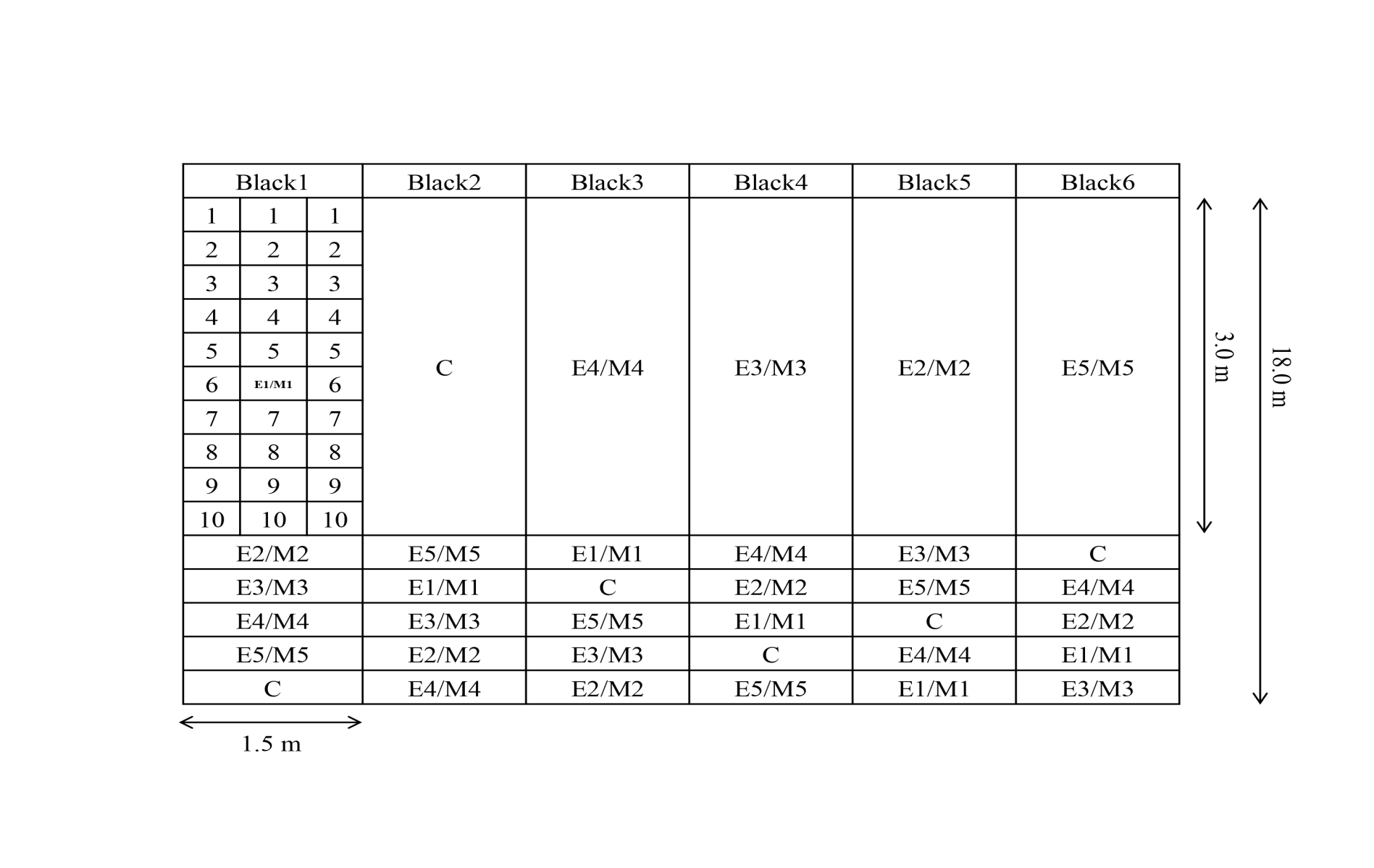
**Fig. S1:** Field layout was prepared according to randomized complete block design (RCBD) in M_1_ generation of *C. annuum* L. Similar pattern was also followed in next generation.

**
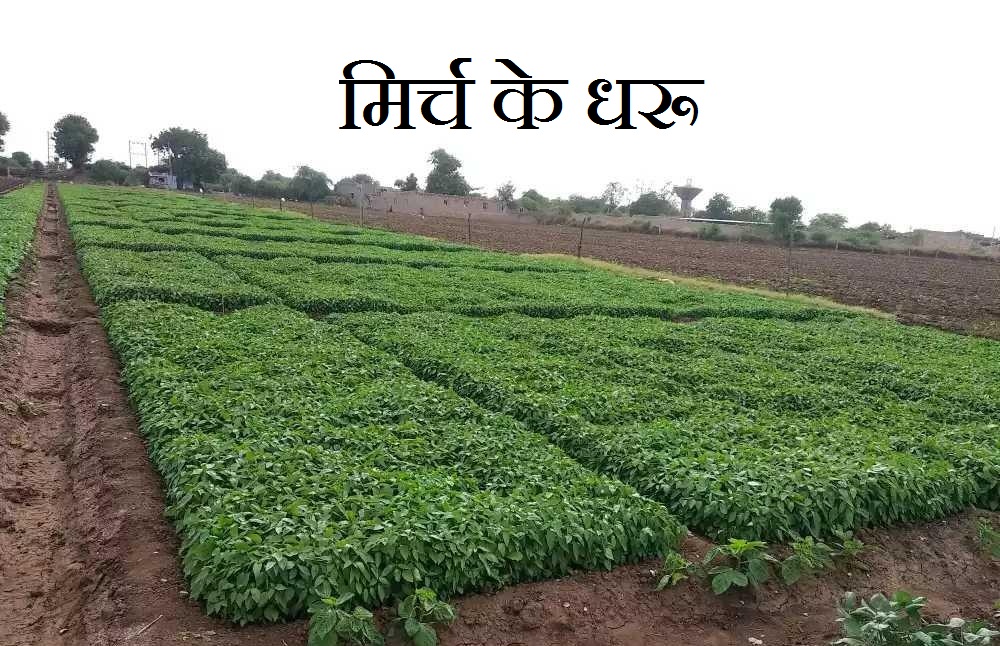
Fig. S2:** *C. annuum* L. growing experimental field.
